# Supplementary material for: manta: a Clustering Algorithm for Weighted Ecological Networks
Source: mSystems. 2020 Feb 18;5(1):e00903-19. doi: 10.1128/mSystems.00903-19 (PMC7029223; doi:10.1128/mSystems.00903-19)
Supplement: FIG S1 [file mSystems.00903-19-sf001.pdf]

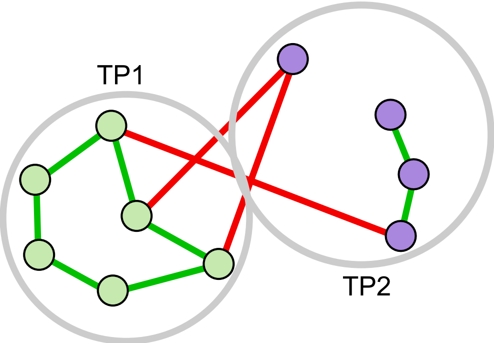

2 clusters with 6 and 4 nodes in each cluster (true positives = TP)

a

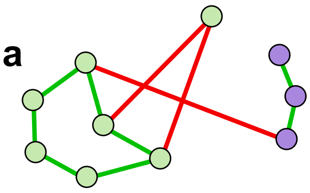

b

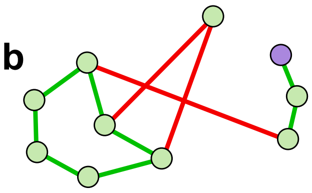

c

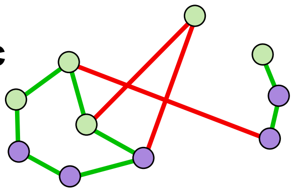

Contingency matrix

	a: A1	a: A2	b: A1	b: A2	c: A1	c: A2
TP1	6	0	6	0	3	3
TP2	1	3	3	1	2	2

Sensitivity (**complex-wise**) value / row sum

	a: A1	a: A2	b: A1	b: A2	c: A1	c: A2
TP1	**1**	0	**1**	0	0.5	0.5
TP2	0.25	**0.75**	**0.75**	0.25	0.5	0.5

Sensitivity (**cluster-wise**) weighted average

a	b	c
**0.9**	**0.9**	**0.5**

Positive predictive value (**complex-wise**) value / column sum

	a: A1	a: A2	b: A1	b: A2	c: A1	c: A2
TP1	**0.86**	0	**0.66**	0	**0.6**	**0.6**
TP2	0.14	**1**	0.33	**1**	0.4	0.4

Positive predictive value (**cluster-wise**) weighted average

a	b	c
**0.9**	**0.7**	**0.6**

Accuracy Geometrical mean

a	b	c
**0.9**	**0.79**	**0.55**

Separation Row frequency * Column frequency

	a: A1	a: A2	b: A1	b: A2	c: A1	c: A2
TP1	0.86	0	0.66	0	0.3	0.3
TP2	0.04	0.75	0.25	0.25	0.2	0.2

Separation Geometrical mean of cluster-wise and column-wise separation

a	b	c
**0.82**	**0.58**	**0.5**
