# Supplementary figures and images for: manta: a Clustering Algorithm for Weighted Ecological Networks
Source: mSystems. 2020 Feb 18;5(1):e00903-19. doi: 10.1128/mSystems.00903-19 (PMC7029223; doi:10.1128/mSystems.00903-19)

**A**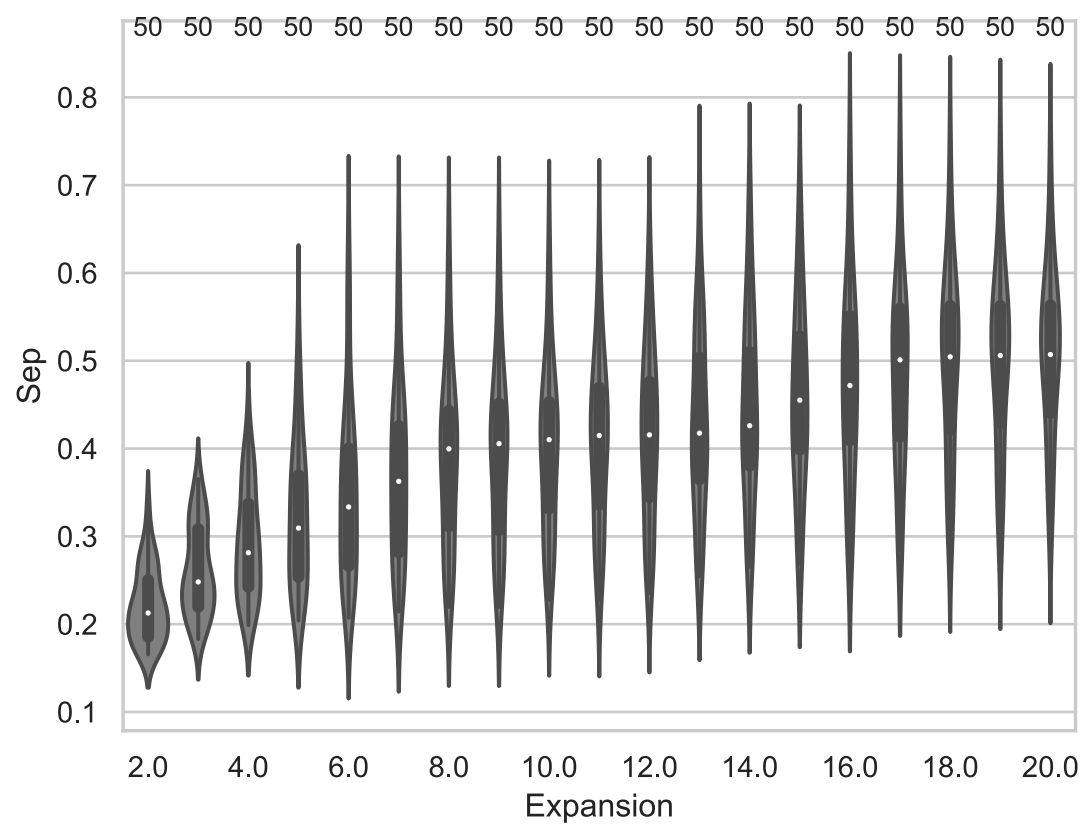**B**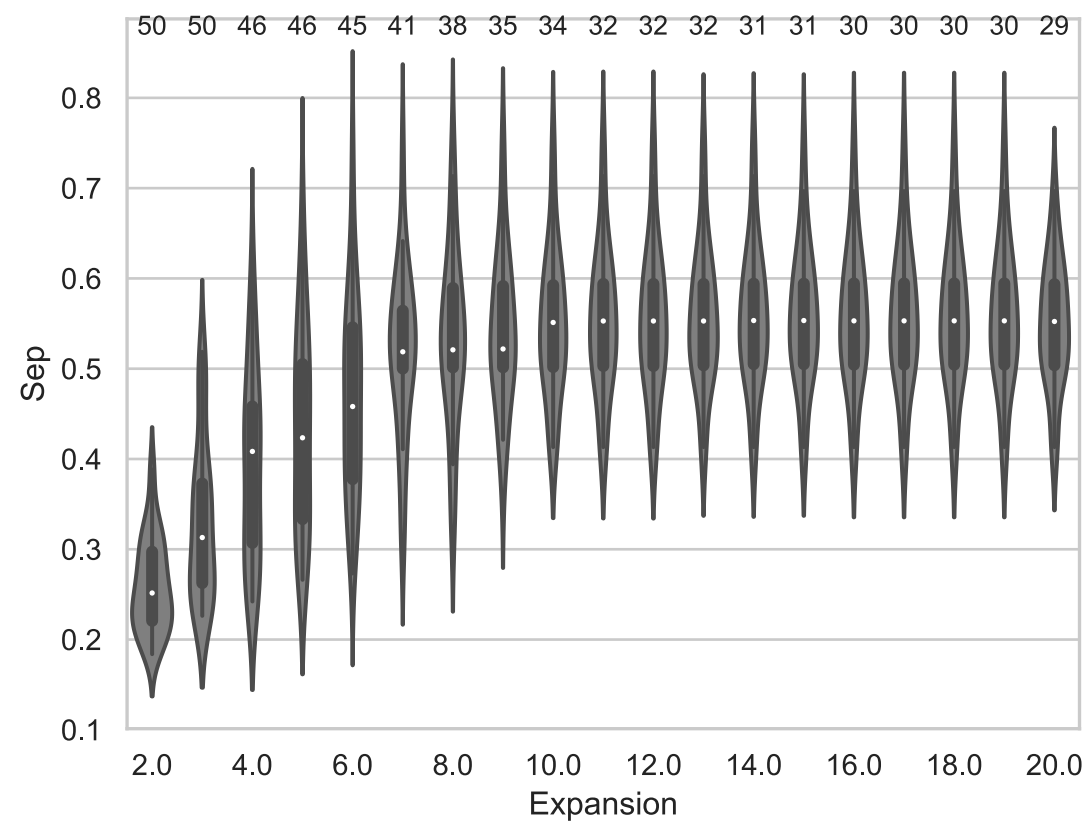**C**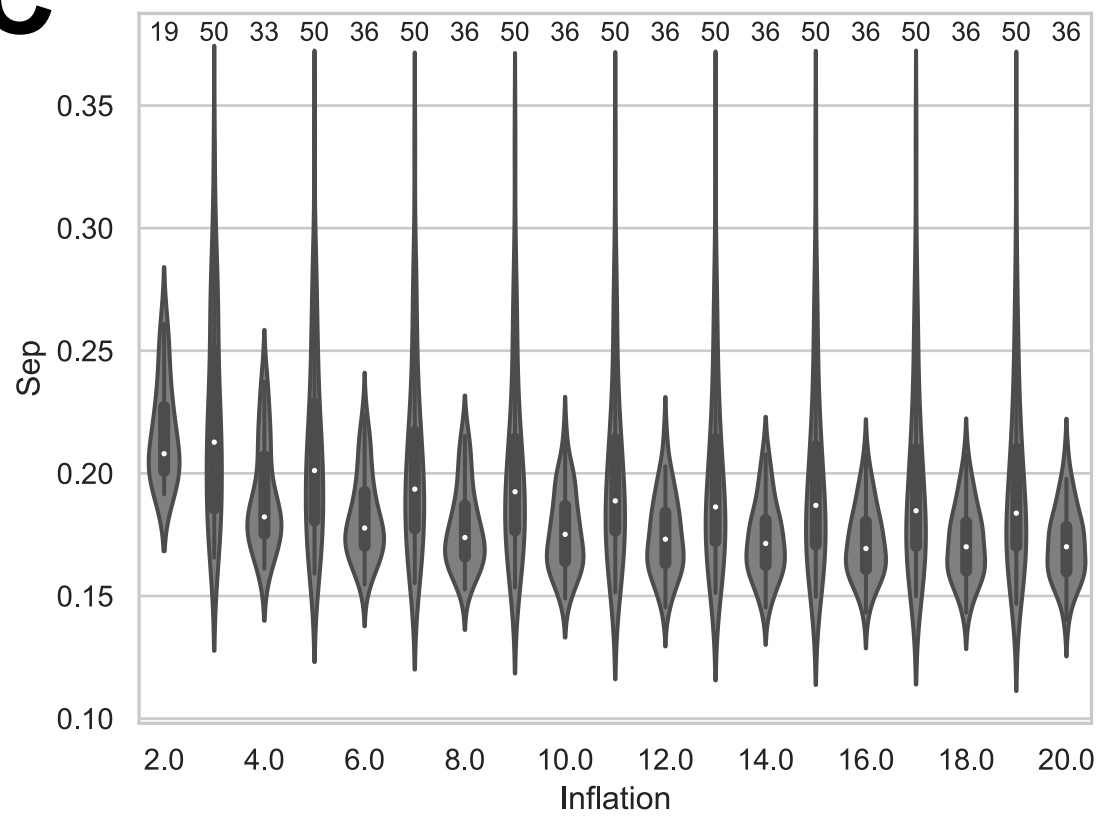**D**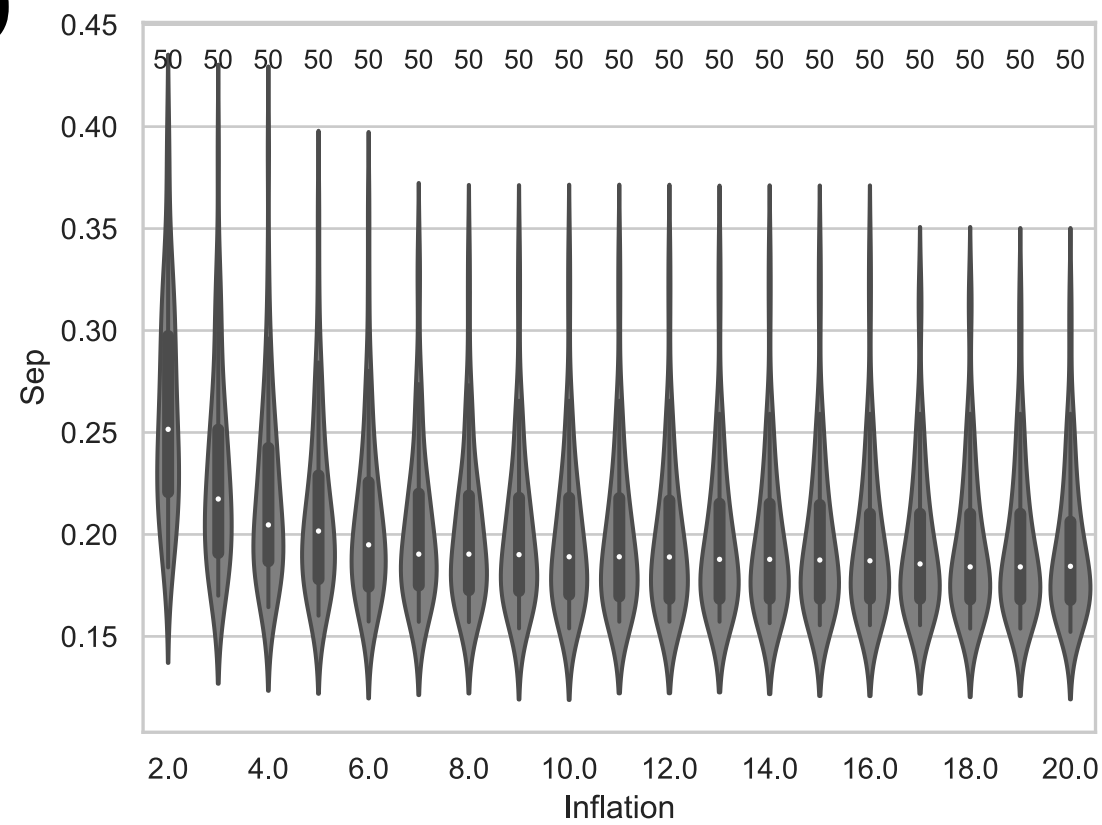

Supplement: FIG S2 [file mSystems.00903-19-sf002.pdf]

**A**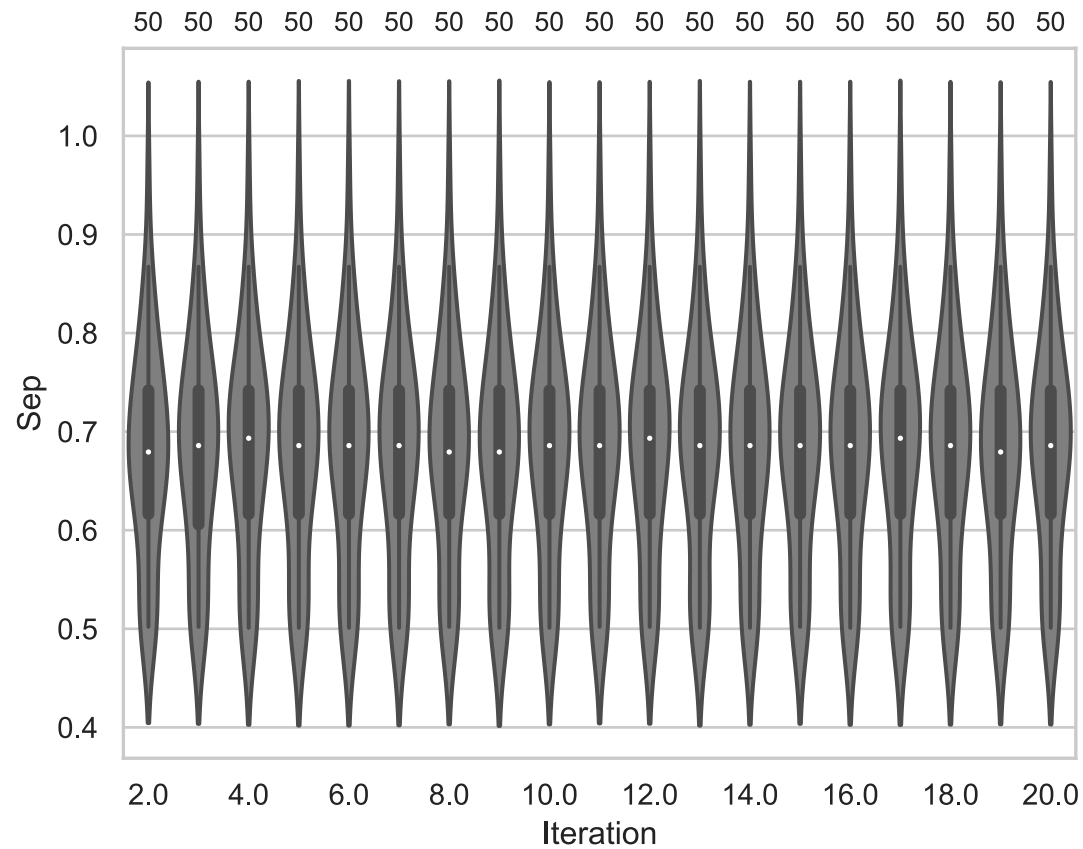**B**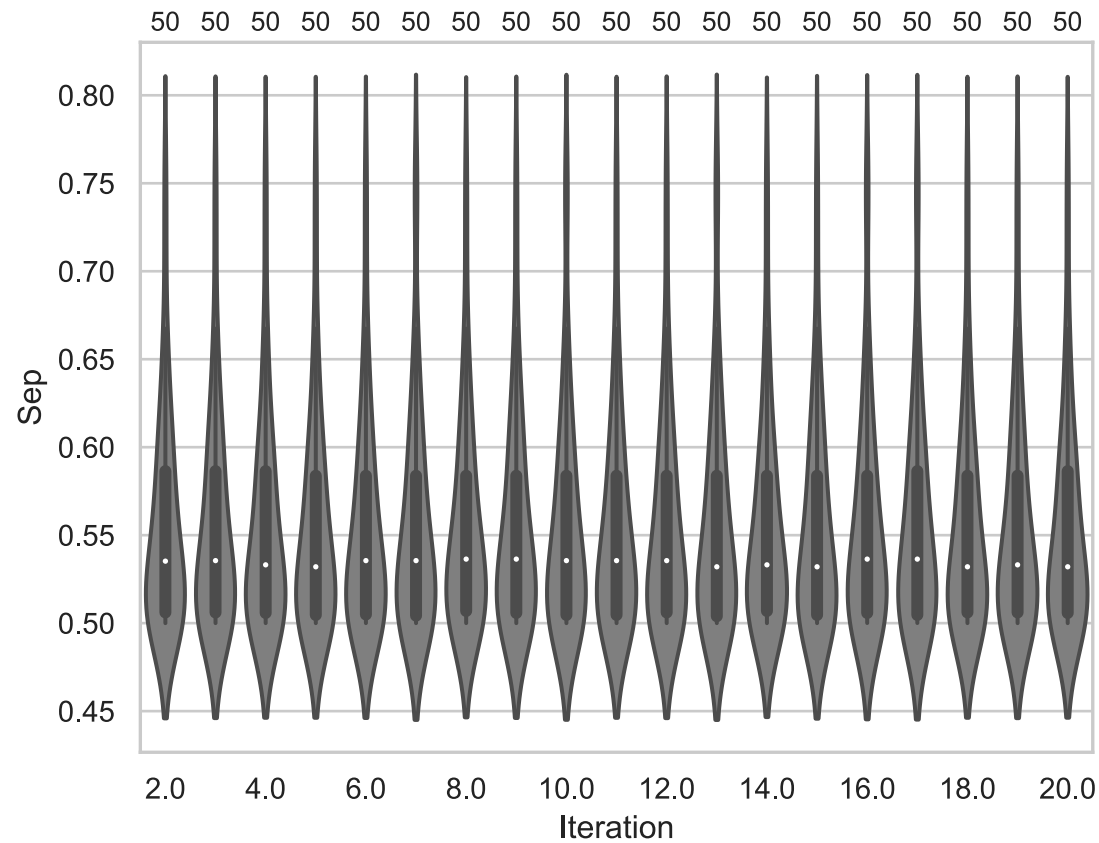

Supplement: FIG S3 [file mSystems.00903-19-sf003.pdf]

**A**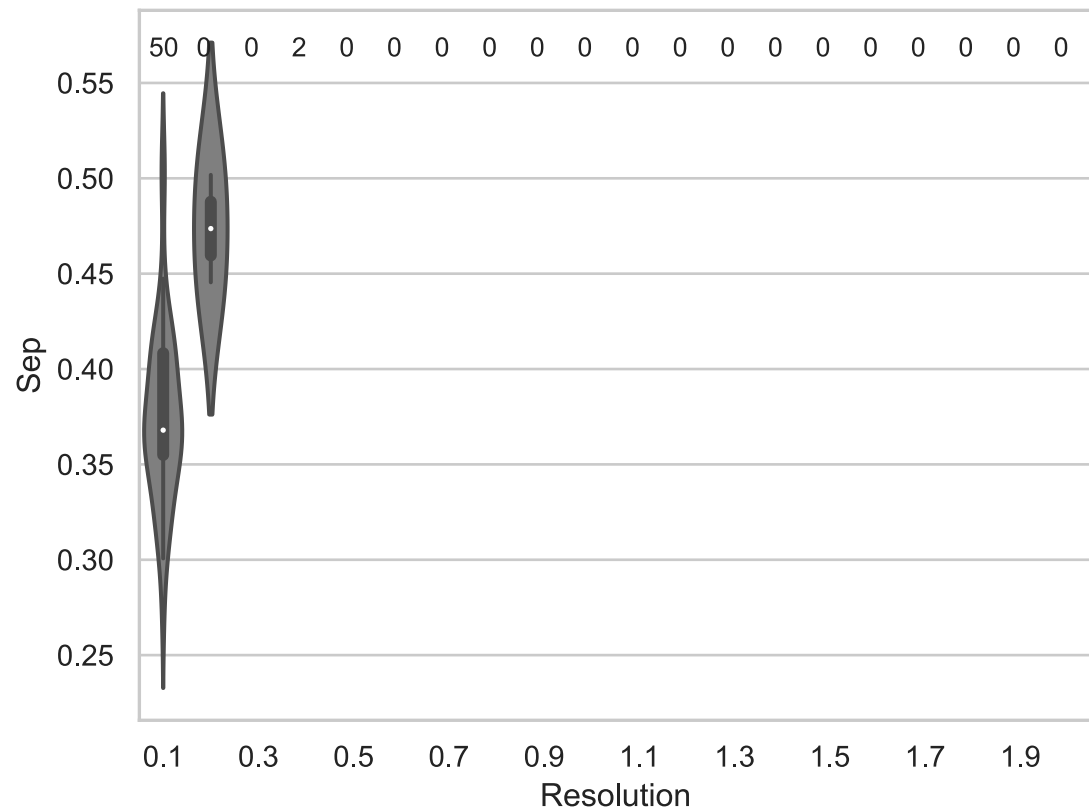**B**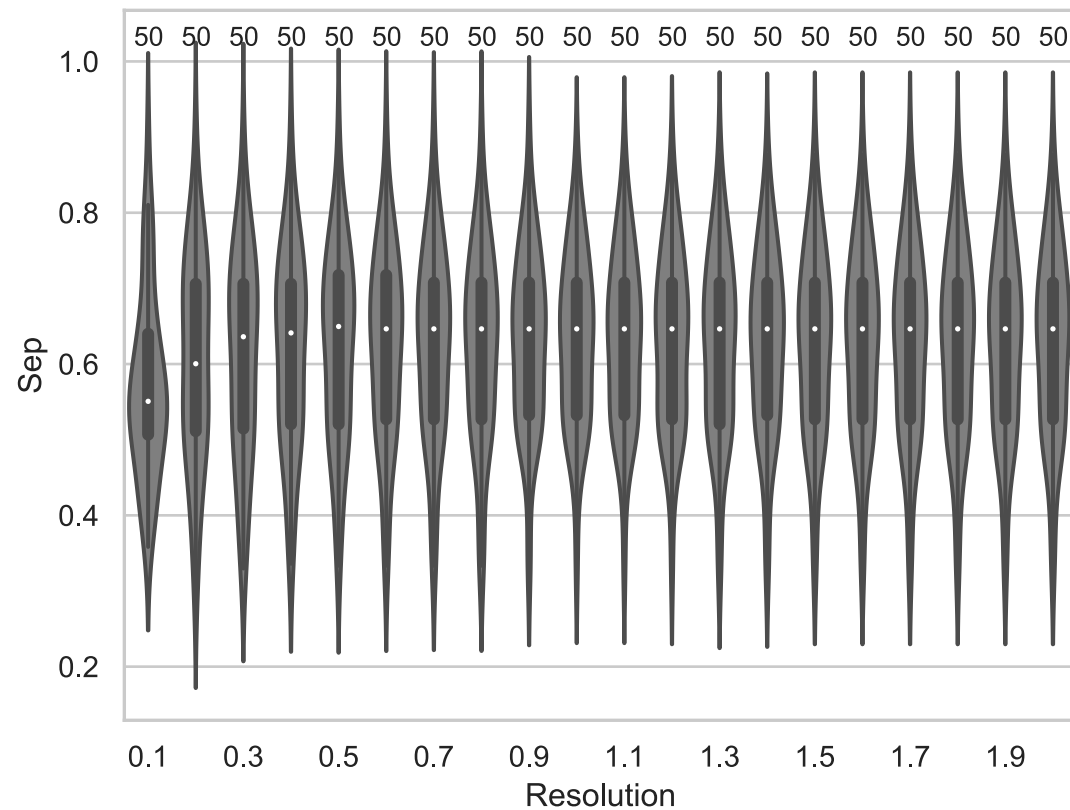

Supplement: FIG S4 [file mSystems.00903-19-sf004.pdf]

**A**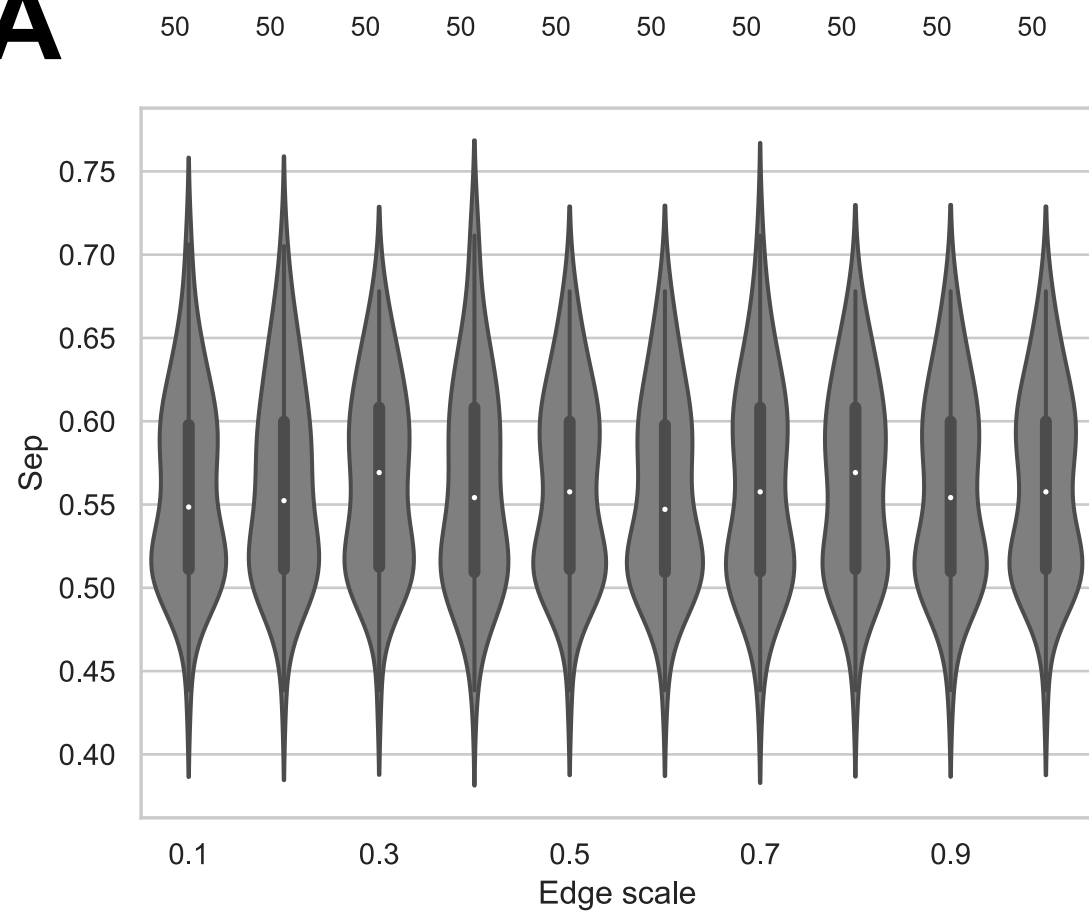**B**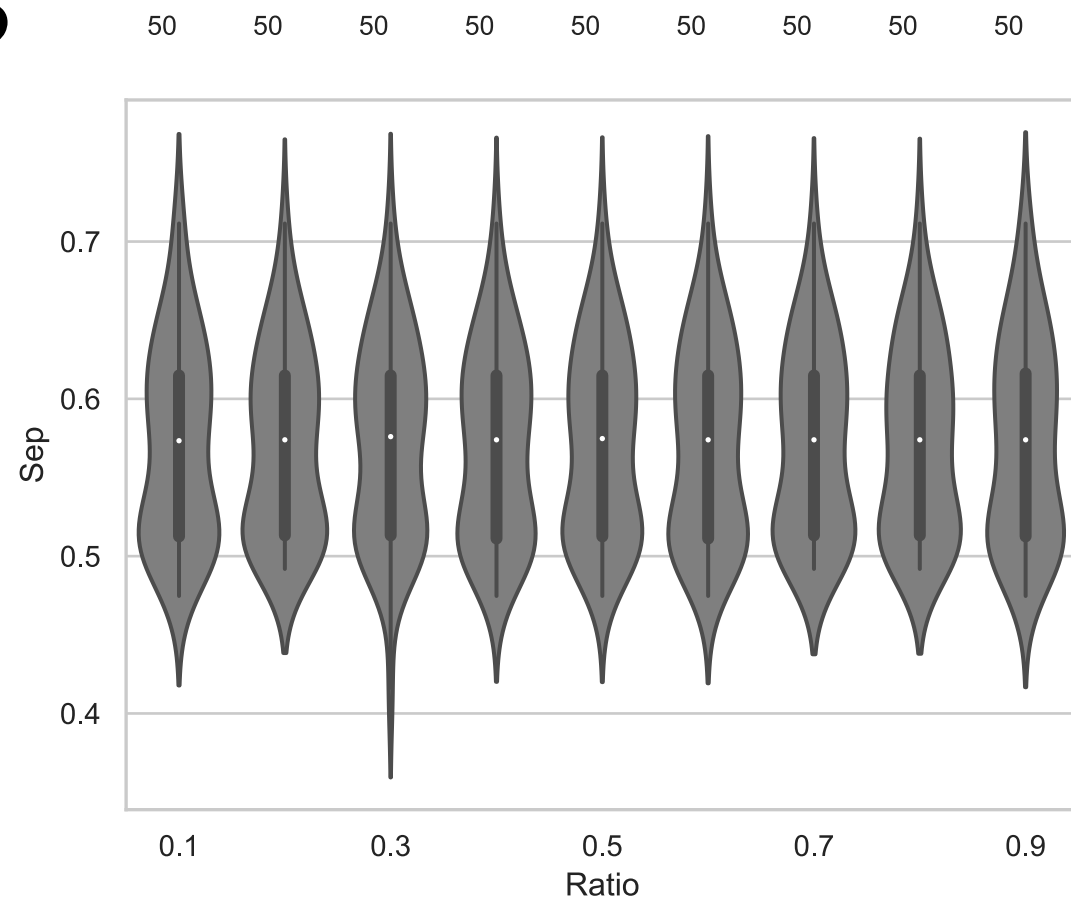**C**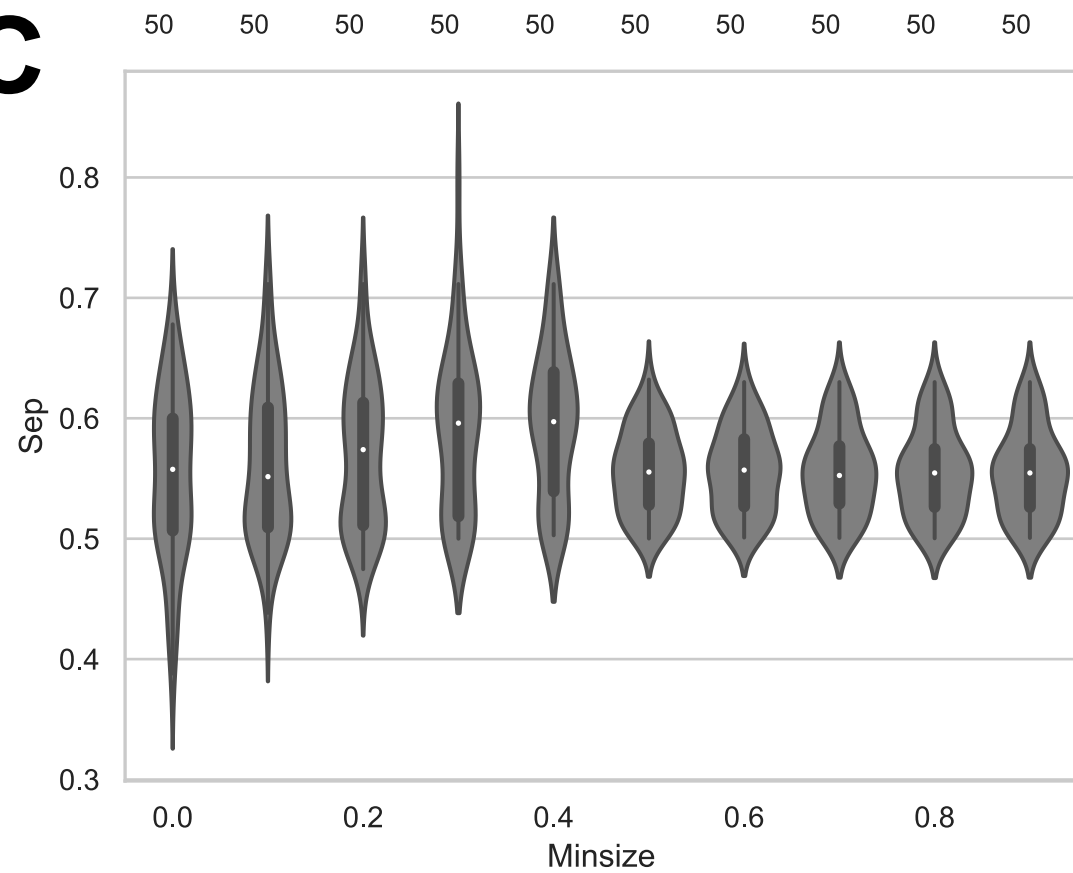**D**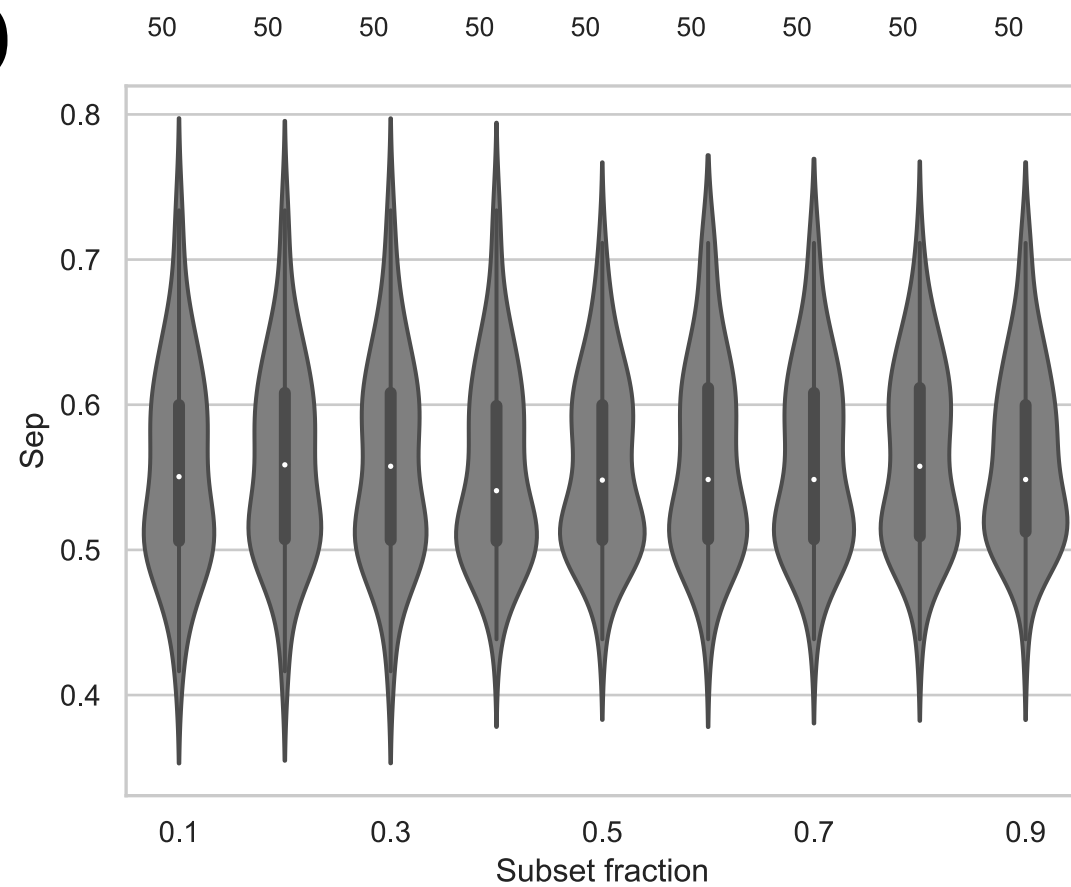

Supplement: FIG S5 [file mSystems.00903-19-sf005.pdf]

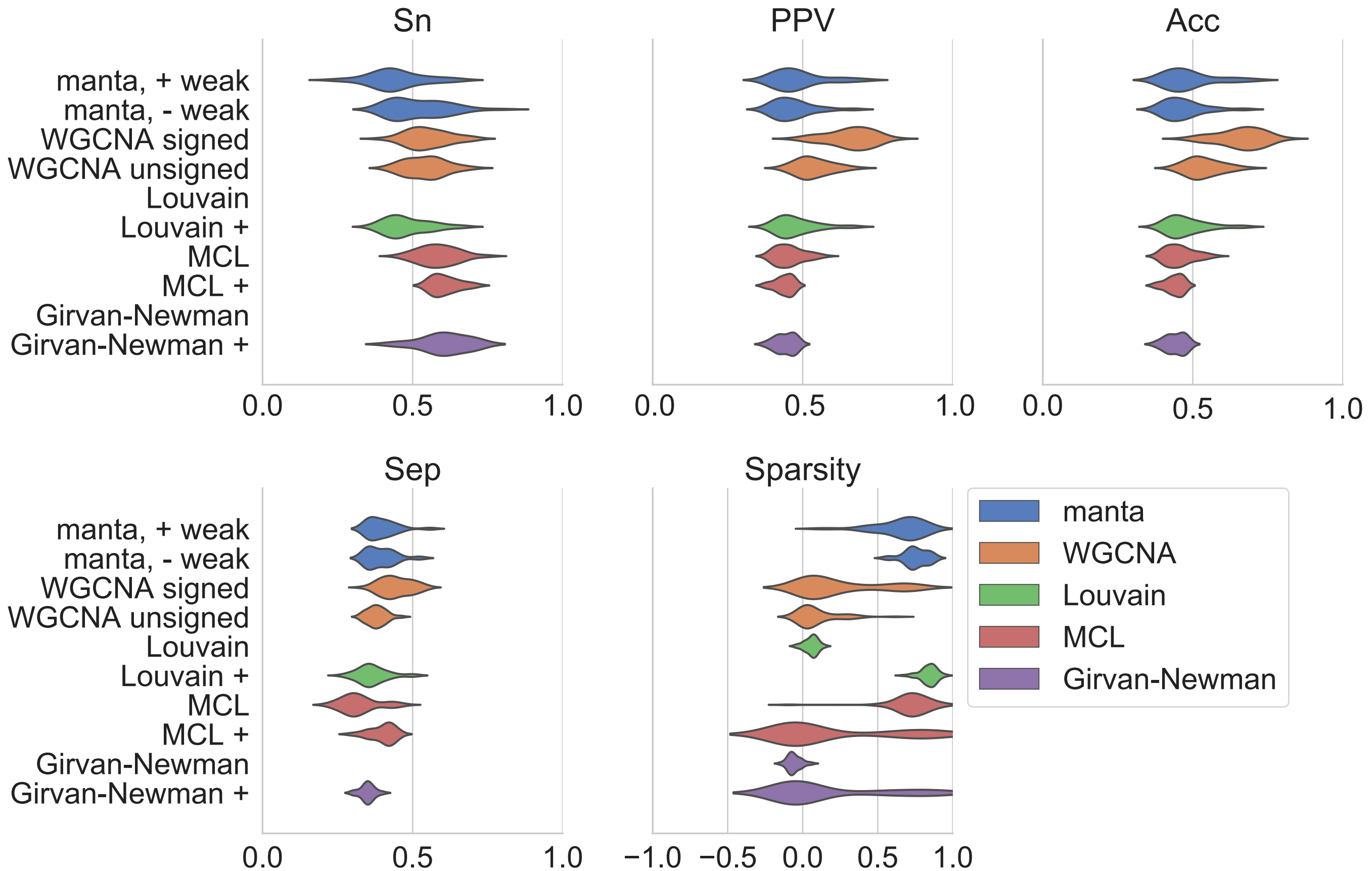

Supplement: FIG S6 [file mSystems.00903-19-sf006.pdf]

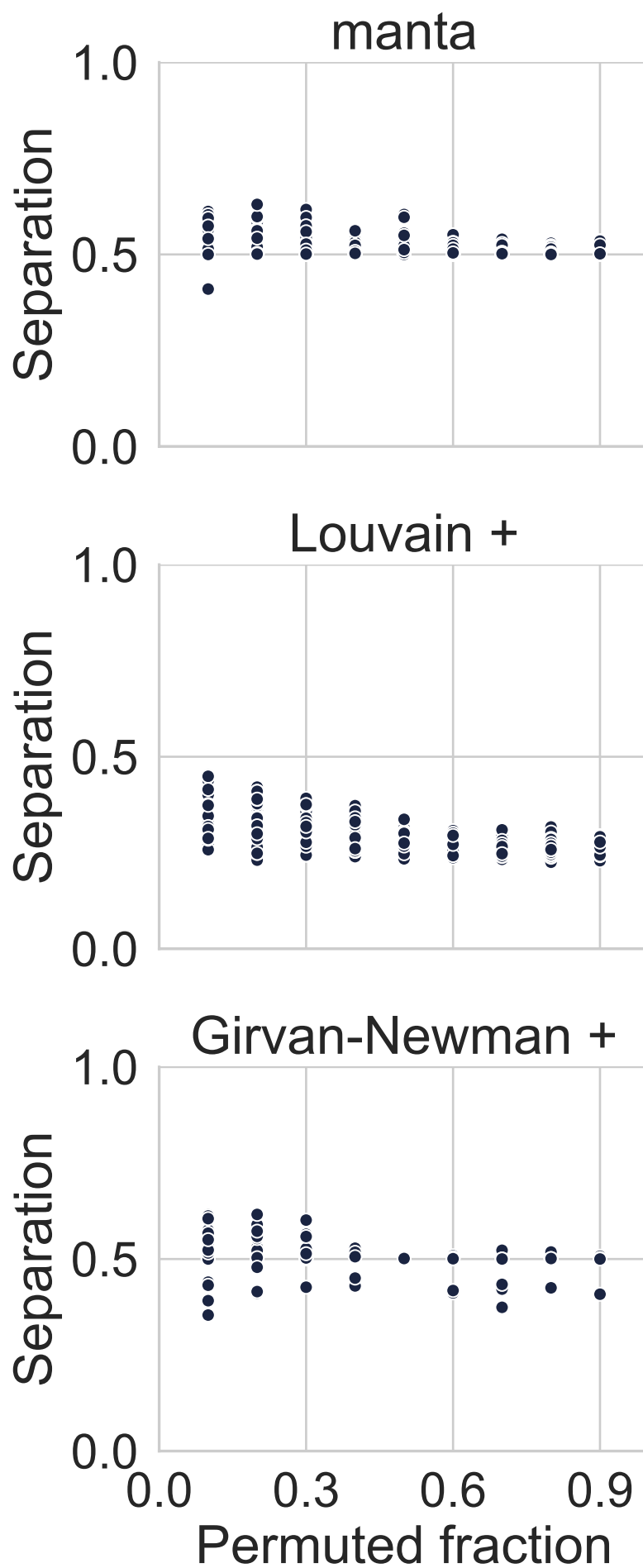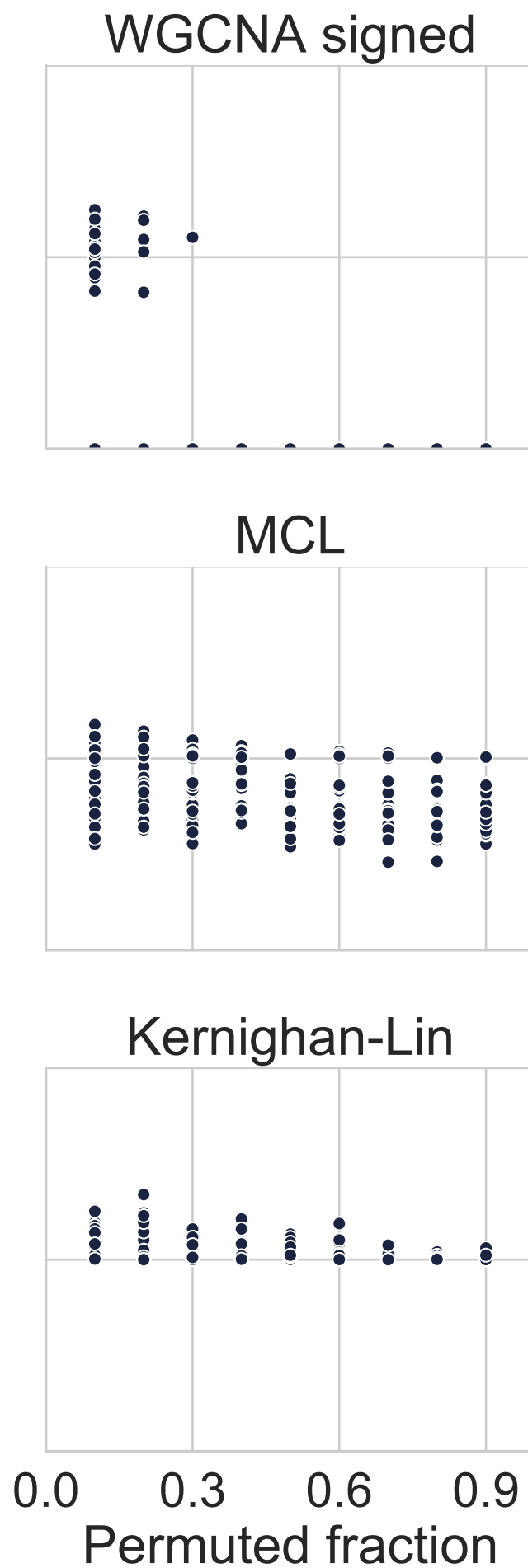

Supplement: FIG S7 [file mSystems.00903-19-sf007.pdf]

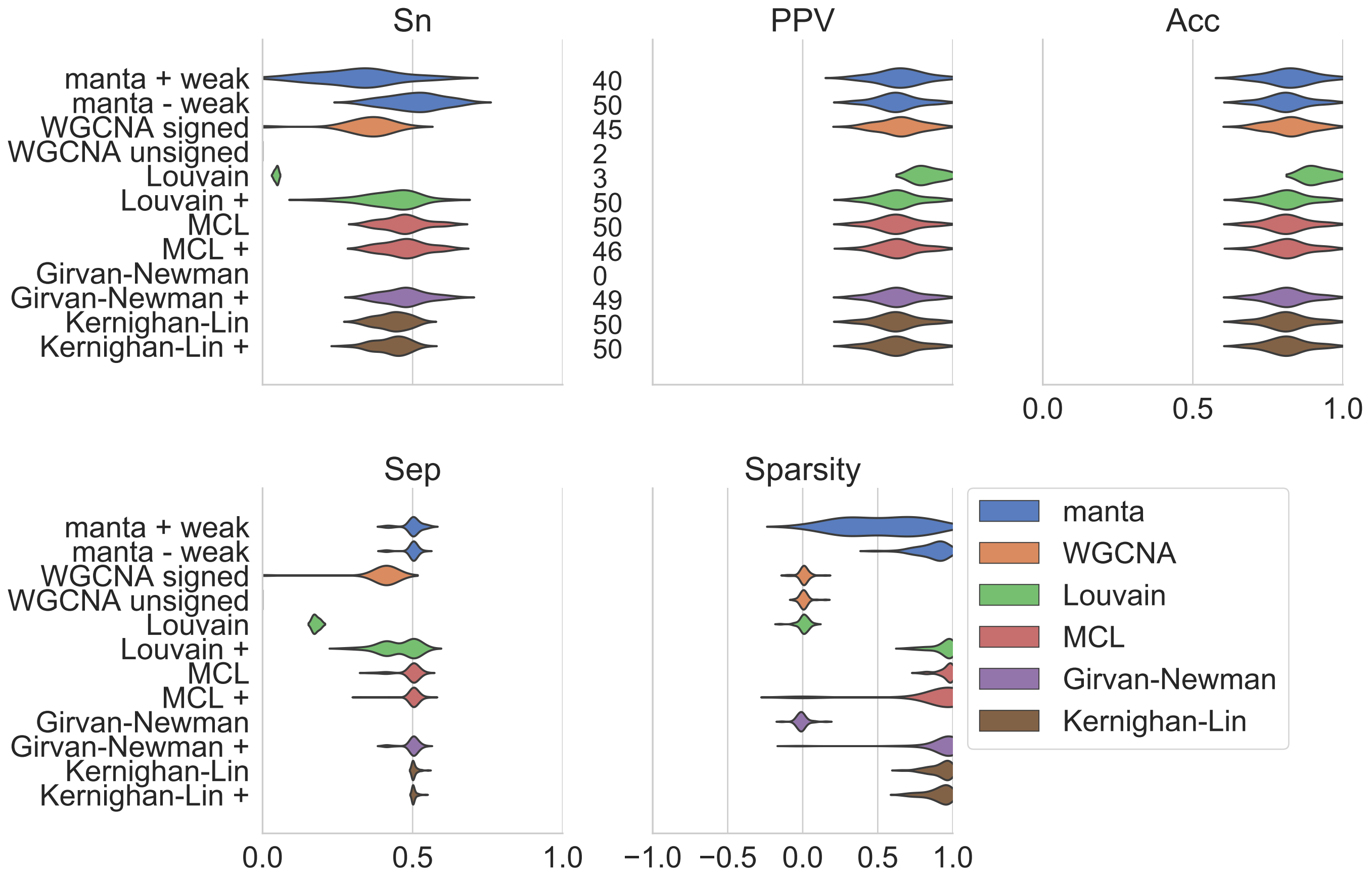

Supplement: FIG S8 [file mSystems.00903-19-sf008.pdf]

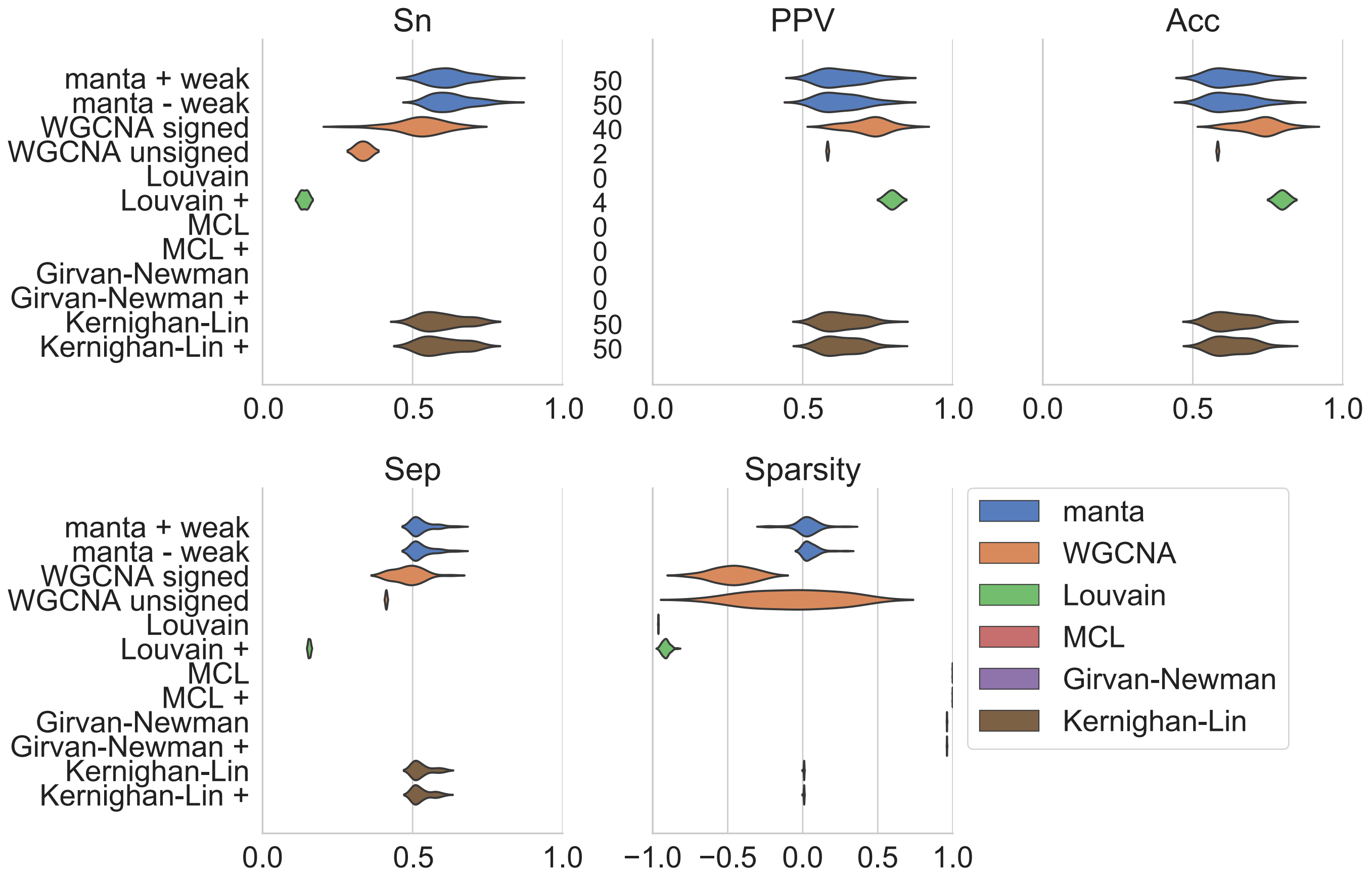

Supplement: FIG S9 [file mSystems.00903-19-sf009.pdf]
